# Supplementary material for: Design of Polysaccharide-Based Nanocomposites for Eco-Friendly Flexible Electronics
Source: Polymers (Basel). 2025 Jun 10;17(12):1612. doi: 10.3390/polym17121612 (PMC12196578; doi:10.3390/polym17121612)
Supplement: Supplementary file 1 [file polymers-17-01612-s001.zip › polymers-3673200-supplementary.pdf]

Supplementary materials

Design of polysaccharide-based nanocomposites for eco-friendly flexible electronics

Gabriela Turcanu [1], Iuliana Stoica[2] , Raluca Marinica Albu[2], Cristian-Dragos Varganici[2] , Mihaela Iuliana Avadanei[2], Andreea Irina Barzic[2,*], Lavinia Curecheriu[1*], Paola Stagnaro[3], Maria Teresa Buscaglia[4*]

[1]"Alexandru Ioan Cuza" University, Faculty of Physics, Blv. Carol I, nr.11, 700506 Iasi, Romania
[2] "Petru Poni" Institute of Macromolecular Chemistry, 41a Grigore Ghica Voda Alley, 700487 Iasi, Romania
[3] CNR-SCITEC, Institute of Chemical Sciences and Technologies "Giulio Natta", National Research Council, 16149 Genoa, Italy
[4] CNR-ICMATE, Institute of Condensed Matter Chemistry and Technologies for Energy, National Research Council, 16149 Genoa, Italy*Correspondence: cosutchi.irina@icmpp.ro (A.I.B.), lavinia.curecheriu@uaic.ro (L.C.), mariateresa.buscaglia@cnr.it (M.T.B.)

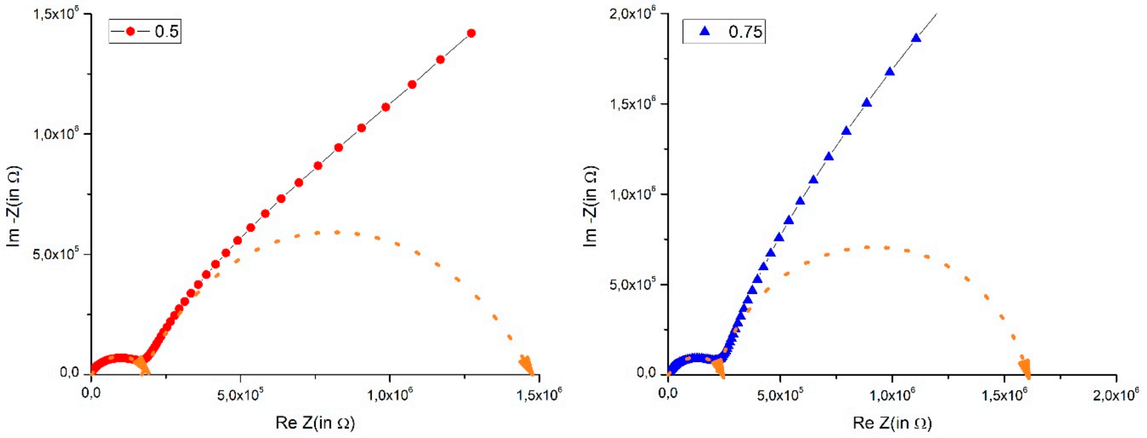

(a) (b)

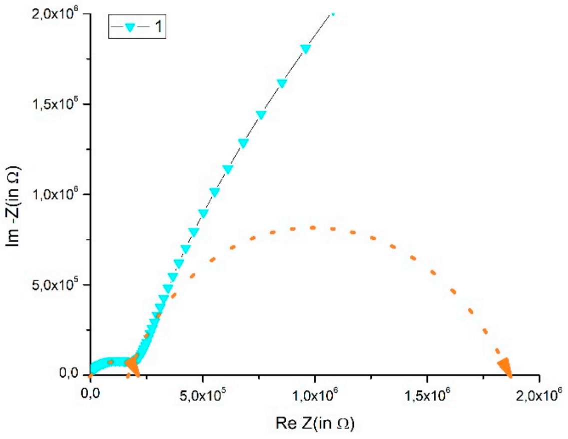

(c)

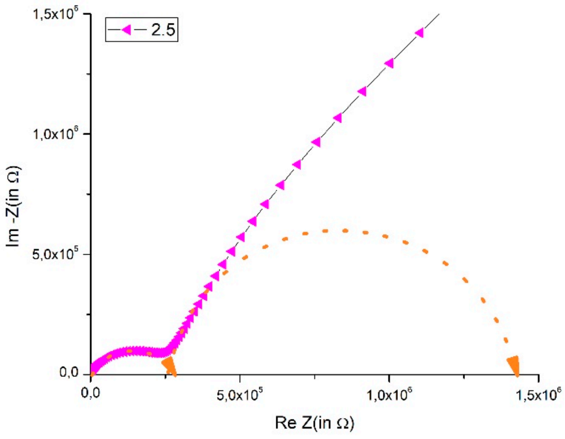

(d)

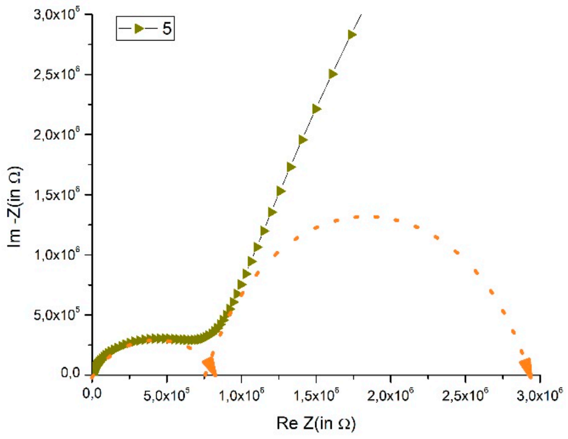

(e)

Figure S1. Fitting experimental Argand diagrams with equivalent circuits for (a) 0.5% BT, (b) 0.75% BT, (c) 1% BT, (d) 2.5% BT, (e) 5% BT
